# Activity of Biocidin^®^ against microbial biofilms

**DOI:** 10.3389/frabi.2025.1692653

**Published:** 2026-01-28

**Authors:** Amy V. Mundanchira, Agnes Wong, Kristen Klos-Maki, Jocelyn Strand, Cláudia N. H. Marques

**Affiliations:** 1 Department of Biological Sciences, Binghamton University, Binghamton, NY, United States; 2 Binghamton Biofilm Research Center, Binghamton University, Binghamton, NY, United States; 3 Biocidin Botanicals, Palm Beach Gardens, FL, United States

**Keywords:** antimicrobials, Biocidin^®^, biofilms, natural derived products, prevention of biofilms

## Abstract

Biofilms—microbial communities—are present throughout the environment and interact with humans as part of the resident microbiome or when causing infection and disease. Antibiotics are commonly used to treat bacterial infections, including those due to biofilms. However, antimicrobial tolerance and resistance are common traits of these microbial communities. Resistance to antimicrobials is now widespread, and the search for alternative treatments, such as plant- or herbal-derived extracts, essential oils, and honey, is on the rise. Here, we investigated the effect of Biocidin^®^, a botanical supplement, on biofilms of *Escherichia coli*, *Staphylococcus aureus*, *Pseudomonas aeruginosa*, *Klebsiella pneumoniae*, and *Candida albicans*. A single (bolus) dose of Biocidin^®^ resulted in a significant decrease (> 2 Log) of biofilm and planktonic populations, while a 24-h continuous dose of 25% and 50% Biocidin^®^ led to a typical biphasic killing curve, with the latter concentration resulting in biofilm eradication of *P. aeruginosa*, *S. aureus*, and *E. coli*. Exposure to sub-inhibitory concentrations of Biocidin^®^ did not affect biofilm viability. Results from this work have implications for the use of Biocidin^®^ as a treatment for biofilm-associated infections and as a supplement in natural medicine.

## 1 Introduction

The development and discovery of antimicrobials increased mostly until the 1960s (Powers, 2004)—the “golden era” of antimicrobial development. It was then thought that infectious diseases were under control (Coates et al., 2002). However, microorganisms can develop resistance through spontaneous mutation or by acquiring DNA from already resistant strains, contributing to widespread antimicrobial resistance (Lipsitch, 2001). In addition to increasing resistance, approximately 70% of infections, including most chronic infections and healthcare-associated infections (HAIs), are due to microbial biofilms (Sharma et al., 2023).

Biofilms constitute the major proportion of bacterial biomass in nature (De Kievit et al., 2001). Colonization and infection occur when microorganisms adhere to a specific surface and produce a matrix enveloping the anchoring microorganisms (the biofilm layer) (Costerton et al., 1994). Microbial biofilms are dynamic communities of cells adherent to inert (abiotic) or living surfaces that are enclosed in a self-produced polymeric matrix (Costerton et al., 1994; Costerton et al., 1995) and react to stimuli in a coordinated manner via intercellular and intracellular communication (Stoodley et al., 2002). The structure of a biofilm protects the cells and enables their survival in hostile environments (Costerton et al., 1999; Kobayashi, 2001). All biofilms share common features: they have a three-dimensional structure and reduced susceptibility to antimicrobials, creating challenges in clinical settings (Zegans et al., 2002). Biofilms are up to 1,000 times less susceptible to antimicrobials compared to their planktonic counterparts (Brown and Gilbert, 1993). However, when bacteria disperse from a biofilm, antimicrobial sensitivity is restored (Stewart, 2002).

In the last two decades, natural products have been explored as alternatives to available antimicrobial treatments. The use of natural products to treat infections is not new, as it has been described in many civilizations, including Mesopotamia and Egypt (Cimino et al., 2021). Most naturally derived products are isolated from plants (Verpoorte et al., 2005). Consequently, validation of their efficacy presents several challenges, ranging from product isolation to loss of concentration during product fractionation (Cos et al., 2006). Despite these setbacks, numerous studies on the antibiofilm activity of naturally derived products have been published in the last two decades, including research on various plants and fruits such as raspberry (Dutreix et al., 2018), cranberry (Bonifait and Grenier, 2010; Girardot et al., 2014), Gentiana lutea (Karalija et al., 2021), and garlic (Bakri and Douglas, 2005; Bjarnsholt et al., 2005; Shuford et al., 2005; Harjai et al., 2010).

In this work, we sought to better understand the effect of Biocidin^®^ on microbial biofilms. Biocidin^®^ is a botanical supplement composed of herbal and oil extracts, including bilberry fruit extract, grape seed extract, shiitake mushroom extract, goldenseal root, noni fruit extract, garlic bulb, white willow bark, milk thistle seed, *Echinacea* purpurea herb extract, Echinacea angustifolia root, raspberry fruit, black walnut hull, black walnut leaf, lavender oil, oregano oil, galbanum oil, tea tree oil, fumitory aerial parts extract, and *Gentiana* lutea root (Biocidin-Scientific Validation of botanical ingredients Bilberry extract (Vaccinium myrtillus)). The exact concentrations of each component are proprietary. This botanical supplement has previously been reported to have a broad spectrum of antimicrobial activity, including efficacy against *Borrelia burgdorferi in vitro* (Karvonen and Gilbert, 2019), treatment of *Molluscum contagiosum* infection (van der Wouden et al., 2017), reduction of pathogenic bacterial overgrowth in the oral cavity (Ebrahimian et al., 2025), efficacy in small intestinal bacterial overgrowth (Min et al., 2024), and maintenance of a balanced microbiome (van der Wouden et al., 2017).

Overall, we found a significant decrease (>2 log) in biofilm and planktonic populations upon exposure to a bolus dose of Biocidin^®^, while a 24-h continuous dose of 25% and 50% Biocidin^®^ led to a typical biphasic killing pattern, with the latter concentration resulting in biofilm eradication of *Pseudomonas aeruginosa, Staphylococcus aureus*, and *Escherichia coli*. In addition, exposure to sub-inhibitory concentrations of Biocidin^®^ did not affect biofilm viability. These results have implications for the use of Biocidin^®^ as a treatment for biofilm-associated infections and as a supplement in natural medicine.

## 2 Materials and methods

### 2.1 Bacterial strains and growth conditions

*Escherichia coli* ATCC 11775, *Pseudomonas aeruginosa* ATCC 10752, *Klebsiella pneumoniae* ATCC 10273, *Staphylococcus aureus* ATCC 6538, and *Candida albicans* ATCC 20260 were used in this study. All overnight cultures were grown in brain heart infusion broth (BHI) in shake flasks at 37°C with shaking (220 rpm), unless indicated otherwise.

### 2.2 Minimum inhibitory concentrations

Minimum inhibitory concentrations (MICs) of each microorganism to Biocidin^®^ (Biocidin Botanicals, FL), ciprofloxacin, tobramycin, fluconazole, and ampicillin were determined in 100% BHI at 37°C after a 24-h incubation. MICs were evaluated in 96-well plates using standard published methodologies (Andrews, 2001). Briefly, overnight microbial cultures were standardized to 0.5 McFarland, and subsequently, 20 μL of the standardized culture were introduced into 180 μL of previously prepared serial dilutions of the compound to be tested. Cultures were incubated at 37°C for 24 h. The MIC was defined as the lowest concentration of the agent that inhibited visible microbial growth. Three biological replicates were performed per condition.

### 2.3 Biofilm cultures

Microbial biofilms were developed in 24-well plates in a semi-batch system, as described previously (Davies and Marques, 2009; Amari et al., 2013; Marques et al., 2015). Briefly, overnight cultures were standardized to an optical density at 600 nm (OD_600_) of 1.0 in 10% BHI, and 0.9 mL were used to inoculate each well. Following 1 h of incubation at 37°C with shaking (200 rpm), the medium was replaced with 0.7 mL of fresh medium (10% BHI) to remove non-adherent microorganisms. Biofilms were then allowed to develop at 37°C with aeration for 5 days, with the medium replenished every 24 h.

### 2.4 Effect of Biocidin^®^ on established biofilms

The response of mature biofilms to Biocidin^®^ was assessed following 5 days of growth. The volume in each well was maintained at 0.7 mL throughout the experiment. On day 5, the medium was replaced with 0.7 mL of solutions containing the different antimicrobials or controls (medium alone). Biofilms were exposed to Biocidin^®^ under the following conditions:

- Exposure of 5-day biofilms to 0%, 25%, 50%, 75%, or 100% Biocidin^®^ (in saline) for 4 h. Controls consisted of saline alone (carrier solution).
- Exposure of 5-day biofilms to 25% and 50% Biocidin^®^ (in 10% BHI) for 24 h, with sampling at 1, 3, 5, and 24 h. Controls consisted of 10% BHI alone (carrier solution).
- Exposure of 5-day biofilms to 5% and 10% Biocidin^®^ for 5 days in 10% BHI, with sampling at 24-h intervals. Controls consisted of 10% BHI alone (negative control) and a known antibiotic or antifungal (positive control). Media and treatments were replenished every 24 h.

For conditions (i) and (ii), following each exposure, planktonic cells (culture medium containing suspended cells) and biofilm cells (attached cells) were collected separately. For each sample, the medium containing planktonic cells was transferred into a microcentrifuge tube, homogenized for 20 s using a Tissue-Tearor homogenizer, serially diluted, plated onto BHI agar, and incubated at 37 °C for 24 h, after which cell viability was quantified as total colony-forming units (CFU). The remaining biofilms were scraped from each well using a cell scraper, resuspended in 1 mL of saline, and processed in the same manner as the planktonic fraction (adapted from (Marques et al., 2005; Marques and Nelson, 2019)).

For condition (iii), biofilm and planktonic samples were collected together and processed similarly to the other conditions to quantify cell numbers. All experiments were performed in at least triplicate.

### 2.5 Statistical analysis

Antimicrobial efficacy was assessed by monitoring cell viability and comparing treated wells with control wells. All conditions were performed in triplicate biological experiments. Samples were tested for normality using the Shapiro–Wilk test, after which a one-way analysis of variance (ANOVA) was performed for multivariate analysis, followed by Tukey’s or Dunnett’s multiple-comparison tests with correction for multiple comparisons, using GraphPad Prism version 6.0a.

## 3 Results

Biocidin^®^ is currently available on the market and has been widely used by the general public, as well as tested in patients (Horowitz and Freeman, 2020). To determine the efficacy of Biocidin^®^ in killing microbial biofilms, it was first necessary to establish the minimum inhibitory concentrations (MICs) of Biocidin^®^, which were found to be 6.25% for *Escherichia coli* and 12.5% for all other microbial species tested (**Table 1**).

**Table 1:** Minimum inhibitory concentrations of Biocidin^®^ against several microbial species.

Bacterial species	Minimum inhibitory concentration (MIC)	
	Average	Standard deviation (SD)
*E. coli* (ATCC 11775)	6.25%	0.2%
*S. aureus* (ATCC 6538)	12.5%	1.1%
*P. aeruginosa* (ATCC 10752)	12.5%	0.6%
*K. pneumoniae* (ATCC 10273)	12.5%	0.5%
*C. albicans* (ATCC 20260)	12.5%	0.3%

### 3.1 A 4-hour bolus dose of Biocidin^®^ is effective in reducing biofilm load

**Figure 1:**
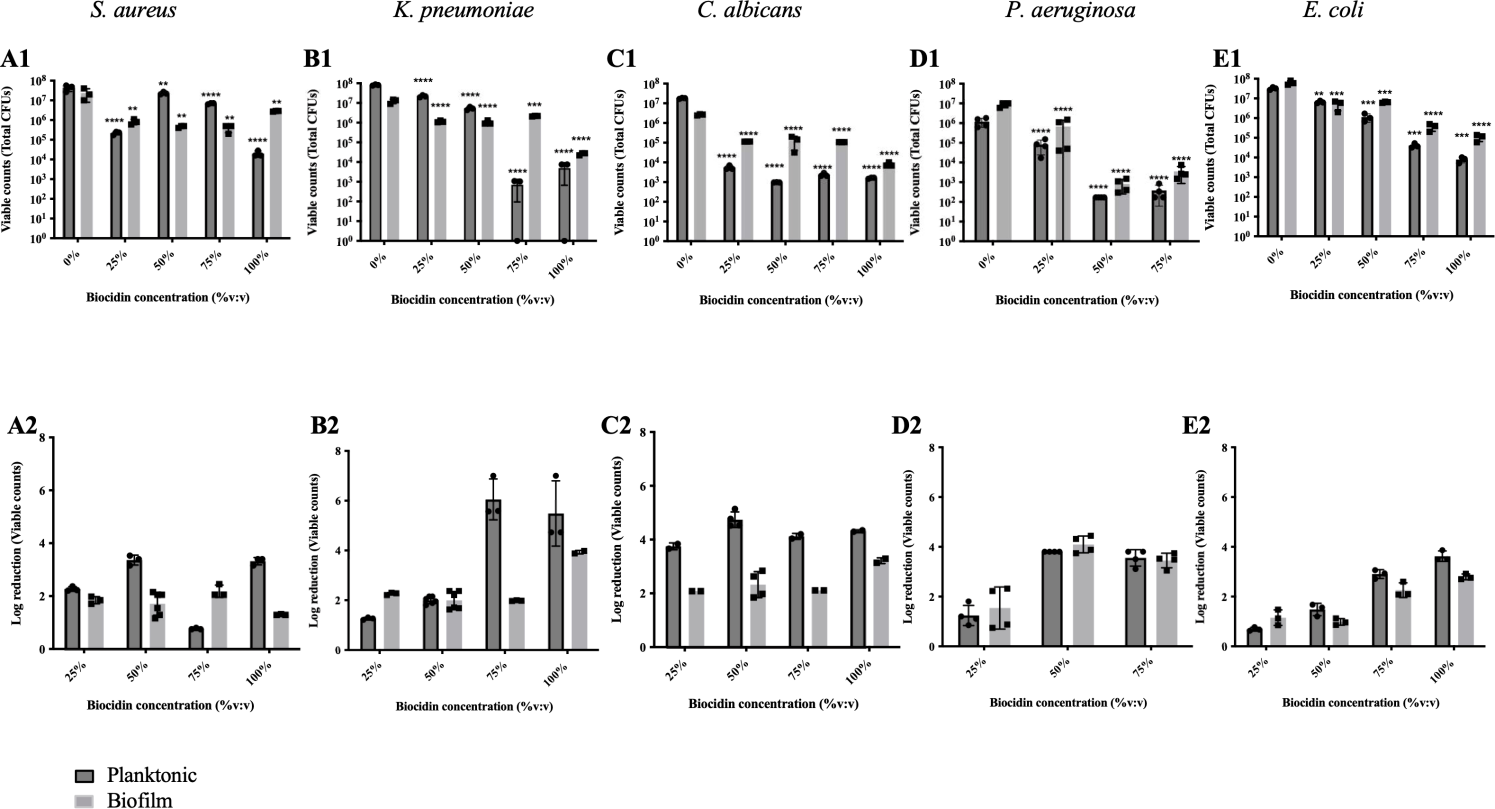
Effect of a bolus concentration of Biocidin^®^ on biofilms. Biofilms were cultured for 5 days and subsequently exposed to Biocidin^®^ at various concentrations for 4h **(A)***Staphylococcus aureus*, **(B)***Klebsiella pneumoniae*, **(C)***Candida albicans*, **(D)***Pseudomonas aeruginosa*, and *E Escherichia coli*. Planktonic and biofilm samples were collected (1). Viable cells (total colony-forming units [CFU]). (2) Log reduction in cell viability. Values represent the mean of at least triplicate experiments, and error bars indicate standard deviation (SD). A two-way analysis of variance (ANOVA) with Tukey’s *post hoc* test was used to determine statistical differences: **p<0.001, ****p<0.0001.

To determine the most efficient concentration of Biocidin^®^ against biofilms, microorganisms were inoculated into wells of a 24-well plate and cultured for 5 days until a steady state was reached. On day 5, biofilms were exposed to concentrations of 25%, 50%, 75%, and 100% Biocidin^®^, diluted in saline, for 4 h (**Figure 1**).

Overall, both biofilm and planktonic cell populations were susceptible to Biocidin^®^, independent of concentration and microorganism tested (**Figures 1A–E**). However, killing efficacy varied by microorganism and population type (biofilm or planktonic). Planktonic cells were more susceptible to Biocidin^®^ than biofilm populations, except for *Pseudomonas aeruginosa* (**Figure 1D**) and *E. coli* (**Figure 1E**), in which susceptibility was similar between the two populations. *Staphylococcus aureus* was the least susceptible microorganism, with a 1–2 log reduction in biofilm viability independent of Biocidin^®^ concentration (**Figure 1A**) and a 2–3 log reduction in planktonic cell viability, except at 75%, which resulted in a 1-log reduction (**Figure 1A2**). *Klebsiella pneumoniae* was the most susceptible microorganism (**Figure 1B**), with planktonic populations decreasing by 6 log upon exposure to 75% and 100% Biocidin^®^, and biofilm populations decreasing by 4 log at 100% Biocidin^®^ (**Figure 1B2**). The effect of Biocidin^®^ on Candida albicans was consistent across concentrations (**Figure 1C**), with planktonic populations decreasing by 4–5 log and biofilm populations by 2–3 log (**Figure 1C2**). For *P. aeruginosa*, the effect of Biocidin^®^ was similar for planktonic and biofilm populations, with killing increasing from 2 log at 25% to 4 log at 50% Biocidin^®^ (**Figure 1D2**). *E. coli* susceptibility increased with increasing Biocidin^®^ concentration, with planktonic killing reaching 3.6 log and biofilm killing reaching 2.8 log at 100% Biocidin^®^ (**Figure 1E2**). A preliminary table of these findings was previously published by Biocidin^®^ (Strand, 2022), albeit without additional interpretation.

### 3.2 Exposure to 25% Biocidin^®^ for 24 h primarily affects bacteria shed from biofilms

**Figure 2:**
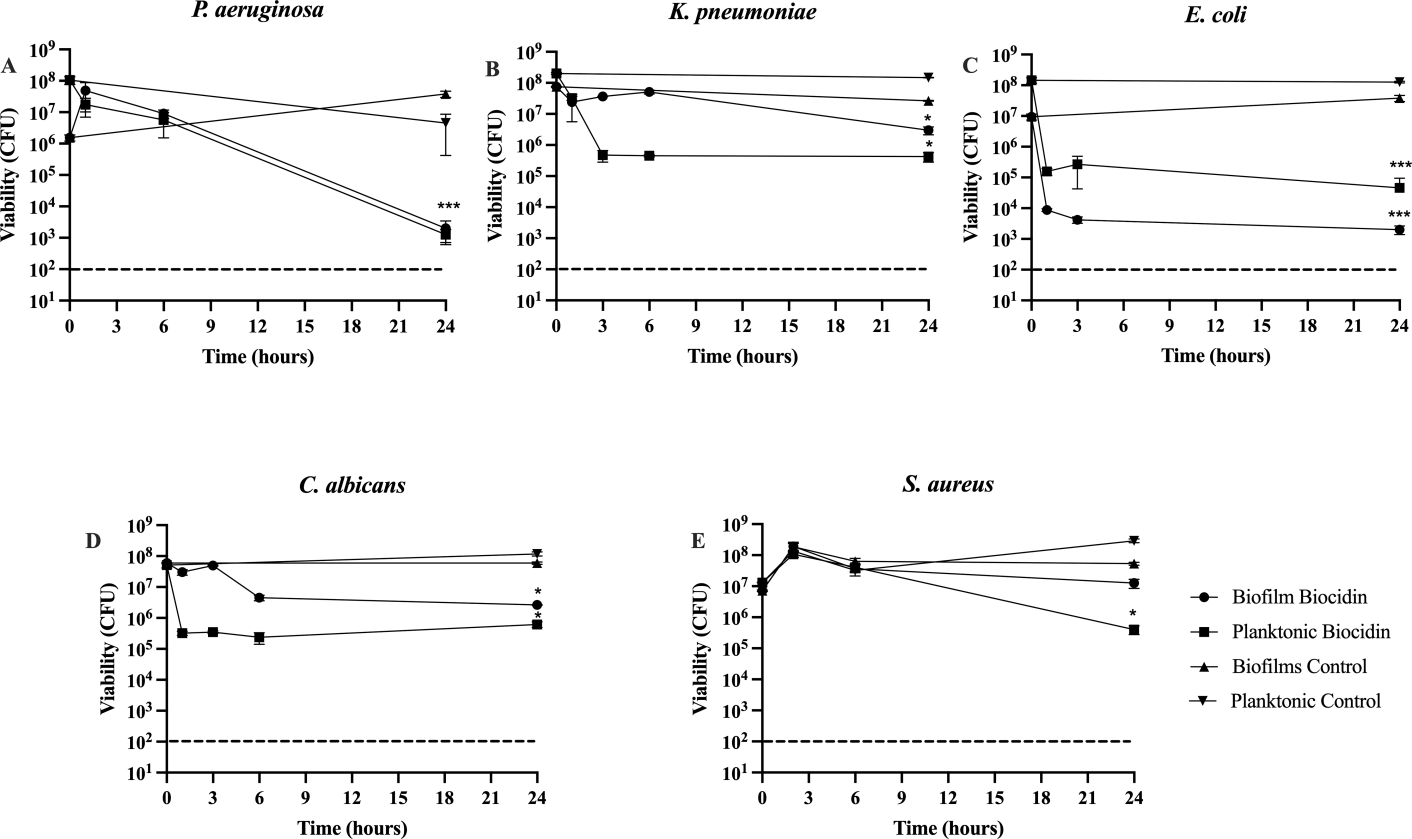
Effect of continuous exposure to 25% Biocidin® on biofilms. Biofilms were cultured for a period of 5 days and subsequently were exposed to 25% Biocidine for 24 hours. **(A)***P. aeruginosa*, **(B)***E. coli*, **(C)***K. pneumoniae*, **(D)***C. albicans*, and **(E)***S. aureus*. Planktonic and biofilm samples were taken at various time intervals within the 24 hours of testing. Values consist of the average of at least triplicate experiments and error bars represent SD. A two-way ANOVA (Analysis of Variance) with Tukey's post-test was used to determine statistical differences * p<0.01, ***p<0.0001. Intermittent line indicates detection limit.

Once it was established that lower concentrations of Biocidin^®^ were effective in killing biofilms when delivered for 4 h (>2 log reduction, ~99%), the lowest effective concentration was selected for extended exposure. Biofilms were exposed to 25% Biocidin^®^ for 24 h, and the viability of biofilm and planktonic cells (shed cells present in the bulk liquid) was monitored at 0, 1, 3, 6, and 24 h (**Figure 2**). Controls consisted of medium alone.

Overall, a reduction in planktonic microbial populations shed from biofilms was observed across all exposures. However, not all biofilm populations were reduced within 24 h (**Figure 2**). *P. aeruginosa* was the most susceptible organism, with planktonic and biofilm populations reduced by 3 log and 5 log, respectively, at 24 h relative to time 0 (**Figure 2A**).

*E. coli* (**Figure 2B**), *K. pneumoniae* (**Figure 2C**), and *C. albicans* (**Figure 2D**) exhibited biphasic killing. *E. coli* biofilm and planktonic populations decreased by approximately 3 log starting at 1 h of exposure (**Figure 2B**). Biofilm populations of *K. pneumoniae* (**Figure 2C**) and *C. albicans* (**Figure 2D**) decreased by 1 log at 24 h, while planktonic populations decreased by approximately 2.5 log beginning at 3 h.

*S. aureus* was the least affected organism following 24-h exposure to 25% Biocidin^®^, with planktonic viability decreasing by 1.5 log (**Figure 2D**).

### 3.3 *S. aureus*, *P. aeruginosa*, and *E. coli* biofilm viability decreases to or below detection limits after 24-h exposure to 50% Biocidin^®^

**Figure 3:**
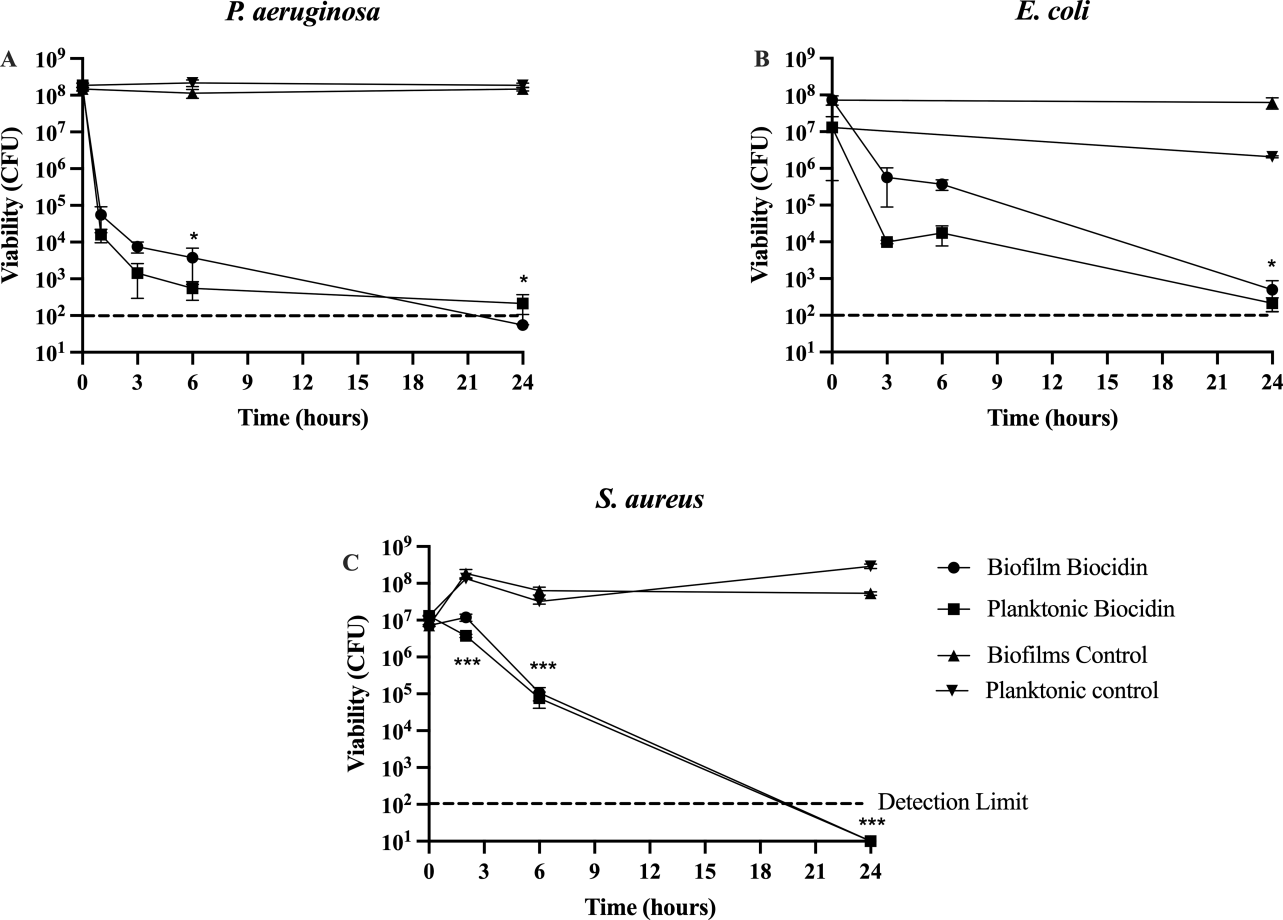
Effect of continuous exposure to 50% Biocidin® on biofilms. Biofilms were cultured for a period of 5 days and subsequently were exposed to 50% Biocidin® for 24 hours. **(A)***P. aeruginosa*, **(B)***E. coli*, **(C)**, *S. aureus*. Planktonic and biofilm samples were taken at various time intervals within the 24 hours of testing. Values consist of the average of at least triplicate experiments and error bars represent SD. A two-way ANOVA (Analysis of Variance) with Tukey's post-test was used to determine statistical differences * p<0.01, **p<0.001, ***p<0.0001. Intermittent line indicates detection limit.

Given the promising results from bolus exposure to various Biocidin^®^ concentrations, we were surprised to find that continuous exposure to 25% Biocidin^®^ did not result in killing above 99.9% (**Figure 2**). Therefore, the effect of 50% Biocidin^®^ was tested against *S. aureus, P. aeruginosa*, and *E. coli* (**Figure 3**).

*P. aeruginosa* (**Figure 3A**) and *E. coli* (**Figure 3B**) exhibited biphasic killing, whereas *S. aureus* (**Figure 3C**) showed exponential killing. *P. aeruginosa* biofilm and planktonic populations decreased by 3.5–4 log within 1 h of exposure (**Figure 3A**). Viability continued to decrease significantly (P < 0.001), reaching a plateau by 3 h and falling below the detection limit by 24 h (**Figure 3A**).

*E. coli* biofilms (**Figure 3B**) followed a similar pattern, with biofilm and planktonic populations decreasing by 2–2.5 log, respectively, and reaching the detection limit at 24 h. *S. aureus* biofilm viability did not plateau and was undetectable by 24 h (**Figure 3C**).

### 3.4 Continuous exposure to sub-inhibitory Biocidin^®^ concentrations does not affect the viability of biofilms.

**Figure 4:**
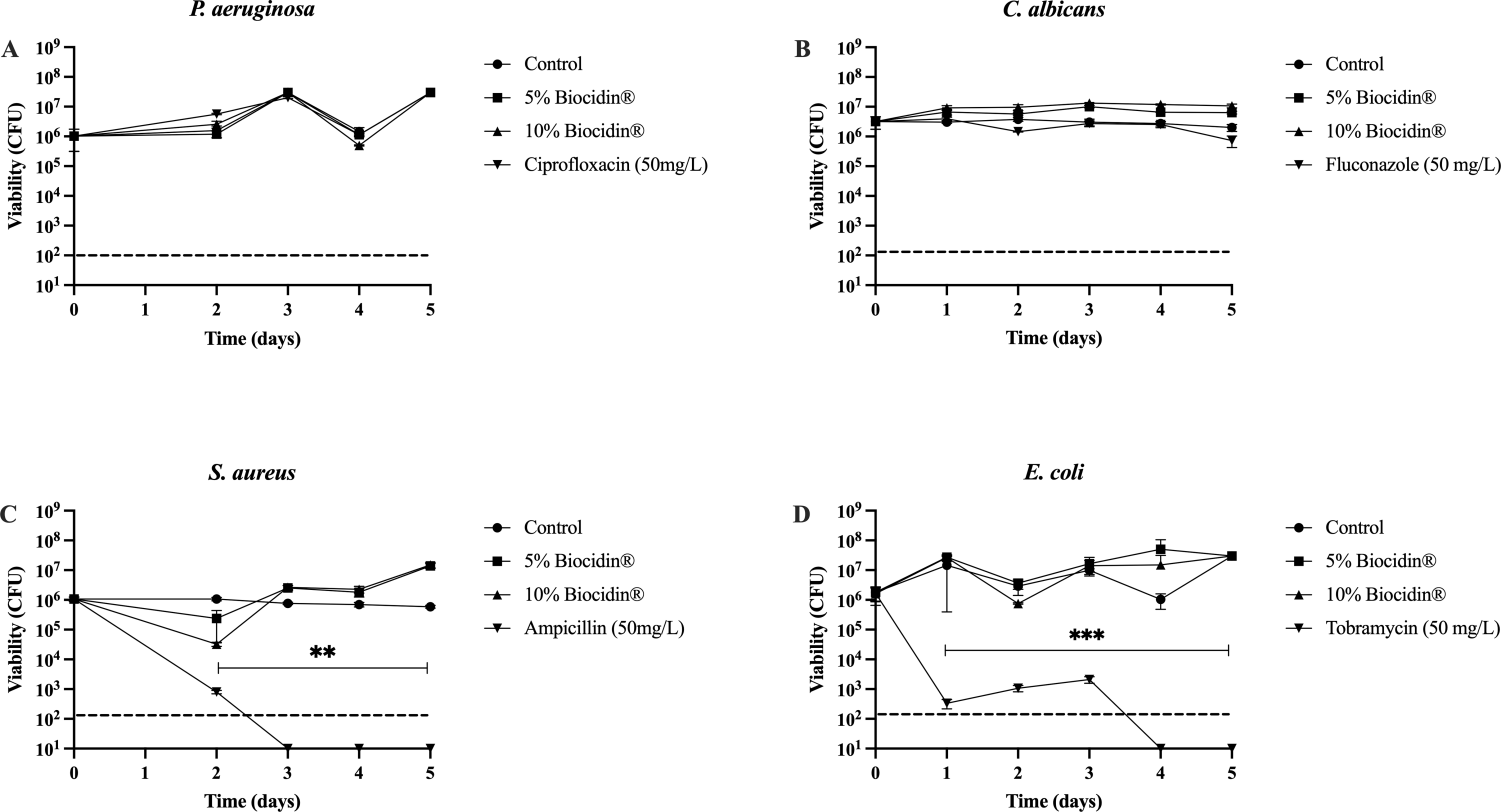
Effect of continuous exposure to sub-inhibitory Biocidin® concentrations on biofilms. Biofilms were cultured for a period of 5 days and subsequently were exposed to 5% and 10% Biocidin® in 10% BHI for further 5 days. Biofilm samples were taken daily. Controls consisted of 10% BHI alone - carrier solution (-) and a known antibiotic or antifungal (+). **(A)***P. aeruginosa*, **(B)***E. coli*, **(C)***K pneumoniae*, **(D)***C. albicans*, and **(E)***S. aureus*. Values consist of the average of at least triplicate experiments and error bars represent SD. A two-way ANOVA (Analysis of Variance) with Tukey's post-test was used to determine statistical differences **p<0.001, ***p<0.0001. Intermittent line indicates detection limit.

To determine whether Biocidin^®^ concentrations below the MIC affected biofilms, 5-day mature biofilms were exposed to 5% and 10% Biocidin^®^. Antimicrobials known to be effective against each microorganism were used as positive controls, while carrier medium alone served as the negative control. Overall, these Biocidin^®^ concentrations did not significantly affect microbial viability (**Figure 4**).

In *S. aureus*, exposure to 5% and 10% Biocidin^®^ resulted in a slight but not statistically significant increase in viability (P > 0.05) (**Figure 4C**). Ciprofloxacin and fluconazole were not effective in killing *P. aeruginosa* (**Figure 4A**) and *C. albicans* (**Figure 4D**) biofilms, respectively, at the concentrations tested.

## 4 Discussion

Natural products composed of concentrated herbal and oil extracts have been used for many centuries and have been shown to have medicinal benefits, including activity against infections.

The human microbiota is composed of a complex microbial community consisting not only of bacteria but also fungi, viruses, and eukaryotic parasites (Mahowald et al., 2009; Flandroy et al., 2018; O’Sullivan et al., 2019; Kennedy and Chang, 2020; Ruan et al., 2020). These microbial communities are commonly found as biofilms. The microbiota is largely beneficial; however, during disease states and following antimicrobial treatments, microbial communities can become imbalanced, resulting in dysbiosis, in which certain microorganisms become predominant while remaining in biofilm form (Kobayashi et al., 2015; Ochoa-Reparaz et al., 2017; Blicharz et al., 2021; Ancona et al., 2023; Zhan et al., 2023). Biofilms are known for their resilience to antimicrobials due to resistance, tolerance, and/or persistence mechanisms (Brown et al., 1988; Hoyle and Costerton, 1991; Brauner et al., 2016; Balaban and Liu, 2019; Sharma et al., 2023). These characteristics, together with the global rise in antibiotic resistance, have led to increased research into alternative or novel treatment strategies aimed at overcoming reduced antibiotic efficacy (de la Fuente-Nunez et al., 2023). Such strategies include the use of bacteriophages (Barr et al., 2013; Roszak et al., 2022; Biswas et al., 2024), honey (Maddocks and Jenkins, 2013; Albaridi, 2019), and naturally derived products such as plant extracts and oils (Butler and Buss, 2006; Cos et al., 2006; Mahady et al., 2008).

To further explore the potential of natural products as alternative treatments for biofilm-associated infections, we investigated Biocidin^®^, a botanical supplement composed of a mixture of herbal and oil extracts. This supplement was tested against *Escherichia coli*, which is responsible for a variety of diseases, including gut-related disorders (Mirsepasi-Lauridsen et al., 2019; Pakbin et al., 2021; Geurtsen et al., 2022); three ESKAPE pathogens—*Pseudomonas aeruginosa*, *Staphylococcus aureus*, and *Klebsiella pneumoniae*—which are major contributors to healthcare-associated infections (HAIs) (Pendleton et al., 2013; Santajit and Indrawattana, 2016; De Oliveira et al., 2020); and *Candida albicans*, which is associated with oral and vaginal thrush, as well as opportunistic infections following antibiotic treatment (Tuss, 1981; Morales and Hogan, 2010; Nobile and Johnson, 2015). Biofilms were exposed to either a bolus treatment or a 24-h continuous treatment with 25% or higher concentrations of Biocidin^®^. In addition, biofilms were continuously exposed to sub-inhibitory concentrations of Biocidin^®^ for 5 days.

Both continuous and bolus exposure of 5-day-old biofilms to Biocidin^®^ resulted in significant killing of biofilms formed by *S. aureus*, *E. coli*, *P. aeruginosa*, *K. pneumoniae*, and *C. albicans* (**Figures 1**–**3**). A 4-h bolus exposure to Biocidin^®^ at concentrations of 25% or higher resulted in a significant (P < 0.01) reduction of ≥2 log in both biofilm and planktonic populations of *S. aureus*, *P. aeruginosa*, *K. pneumoniae*, and *C. albicans* (**Figure 1**). Increased killing was observed following 24-h exposure to 25% Biocidin^®^, which also included *E. coli* (Gram-negative) (**Figure 2**), with the exception of *S. aureus* (**Figure 2E**). Furthermore, continuous exposure of *P. aeruginosa*, *E. coli*, and *S. aureus* biofilms to 50% Biocidin^®^ for 24 h resulted in biofilm killing to the point of eradication or below the detection limit (**Figure 3**).

These findings complement previous reports demonstrating that Biocidin^®^ is effective in killing *Borrelia burgdorferi*, the causative agent of Lyme disease (Karvonen and Gilbert, 2019). The broad-spectrum antimicrobial activity and high efficacy in reducing biofilm viability observed in this study were anticipated based on the herbal composition of Biocidin^®^. Antimicrobial activity has previously been demonstrated for all major components of Biocidin^®^, including bilberry extract (Vučić et al., 2013), grape seed extract (Han et al., 2021; Krasteva et al., 2023), shiitake mushroom extract (Hearst et al., 2009; Kupcová et al., 2018), goldenseal root (Gao et al., 2024), noni fruit extract (Rae et al., [[NoYear]]; Jahurul et al., 2021), garlic bulb (Bakri and Douglas, 2005; Bjarnsholt et al., 2005; Shuford et al., 2005; Harjai et al., 2010; Bhatwalkar et al., 2021), milk thistle seed (Doğan et al., 2022; Kucharska et al., 2024), *Echinacea purpurea* herb extract (Ismali, 2022; Yazdanian et al., 2022; Burlou-Nagy et al., 2023), *Echinacea angustifolia* root (Ismali, 2022), raspberry fruit (Dutreix et al., 2018; Gomathi et al., 2024), black walnut (D’angeli et al., 2021; Ho et al., 2023), lavender oil (Ciocarlan et al., 2021; Mesic et al., 2021; Speranza et al., 2023), oregano oil (Lee et al., 2017; Lu et al., 2018; Hacioglu et al., 2021; Lu et al., 2022), tea tree oil (Kabir Mumu and Mahboob Hossain, 2018; Puvača et al., 2019), and *Gentiana* lutea root (Karalija et al., 2021; Subašić et al., 2022).

In contrast, sub-inhibitory concentrations of Biocidin^®^ did not significantly affect biofilms of *P. aeruginosa*, *S. aureus*, *E. coli*, or *C. albicans*, even after 5 days of continuous exposure (**Figure 4**). This finding suggests that at low concentrations, Biocidin^®^ does not impair microbial viability. However, it may exert effects at the molecular level, potentially reducing microbial virulence—an effect that has been observed for several herbal extracts (Subašić et al., 2022; Křížkovská et al., 2023; Muñoz-Cázares et al., 2023).

Overall, Biocidin^®^ demonstrated significant *in vitro* antimicrobial activity against biofilms formed by *E. coli*, *S. aureus*, *P. aeruginosa*, *K. pneumoniae*, and *C. albicans* when present at concentrations of 25% or higher. Although Biocidin^®^ has been commercially available since 1989 with no reported adverse effects associated with long-term use, cytotoxicity data for these concentrations are currently lacking. Furthermore, limited clinical evidence suggests that Biocidin^®^ may promote tissue healing, as demonstrated in a case report involving a child with *Molluscum contagiosum* and in a case report evaluating gingival and periodontal health (van der Wouden et al., 2017). While Biocidin^®^ is generally regarded as well tolerated at recommended doses, the concentrations required for biofilm eradication in this study exceed those likely achievable within the gastrointestinal tract and other internal organs. However, such concentrations may be attainable in localized oral or topical applications, as supported by a recent case study reporting reduced pathogenic bacterial overgrowth in the oral cavity following Biocidin^®^ use (Ebrahimian et al., 2025).

## Data Availability

The raw data supporting the conclusions of this article will be made available by the authors, without undue reservation.
